# Anchoring Water Soluble Phosphotungstic Acid by Hybrid Fillers to Construct Three-Dimensional Proton Transport Networks

**DOI:** 10.3390/membranes11070536

**Published:** 2021-07-15

**Authors:** Shaojian He, Zhongrui Lu, Wenxu Dai, Kangning Yang, Yang Xue, Xiaoyang Jia, Jun Lin

**Affiliations:** 1 State Key Laboratory of Alternate Electrical Power System with Renewable Energy Sources, North China Electric Power University, Beijing 102206, China; heshaojian@ncepu.edu.cn (S.H.); luzhongrui2020@163.com (Z.L.); dwx903005767@163.com (W.D.); ykn1115226625@163.com (K.Y.); wunaileahappy@163.com (X.J.); 2 State Key Laboratory of Multi-Phase Complex Systems, Institute of Process Engineering, Chinese Academy of Sciences, Beijing 100190, China

**Keywords:** proton transport network, low relative humidity, stable conductivity, polydopamine, graphene oxide, halloysite nanotube, phosphotungstic acid

## Abstract

Phosphotungstic acid (HPW)-filled composite proton exchange membranes possess high proton conductivity under low relative humidity (RH). However, the leaching of HPW limits their wide application. Herein, we propose a novel approach for anchoring water soluble phosphotungstic acid (HPW) by polydopamine (PDA) coated graphene oxide and halloysite nanotubes (DGO and DHNTs) in order to construct hybrid three-dimensional proton transport networks in a sulfonated poly(ether ether ketone) (SPEEK) membrane. The introduction of PDA on the surfaces of the hybrid fillers could provide hydroxyl groups and secondary amine groups to anchor HPW, resulting in the uniform dispersion of HPW in the SPEEK matrix. The SPEEK/DGO/DHNTs/HPW (90/5/5/60) composite membrane exhibited higher water uptake and much better conductivity than the SPEEK membrane at low relative humidity. The best conductivity reached wass 0.062 S cm^−1^ for the composite membrane, which is quite stable during the water immersion test.

## 1. Introduction

Proton exchange membrane fuel cells (PEMFCs) have been considered to be one of the most promising energy conversion devices due to their high energy efficiency and zero-emission [1,2,3,4]. PEMFC, working at high temperatures, has some advantages that may improve its efficiency [5]. However, most of the PEMs (a key component of PEMFCs) are incapable of operating at high temperatures since water evaporating out from the membrane would result in the loss of proton conductivity. Therefore, PEMs that possess high proton conductivity under low relative humidity (RH) are in urgent need in a range of wide applications [6,7,8]. One of the most promising strategies to obtain such PEMs involves the incorporation of phosphotungstic acid (HPW) into the composite membrane, which can retain water under low RH due to the excellent water retention ability of HPW [9,10,11]. However, HPW is easy to leach out from HPW-filled composite membranes due to the high water solubility, which limits its further application [12].

To avoid the leakage of water soluble HPW, various substrates (e.g., carbon nanotubes, sub-micro-porous chitosan, polyvinylpyrrolidone, graphene oxide, and graphitic carbon nitride) were used to anchor HPW [13,14,15,16,17]. In our previous work, imidazole-functionalized halloysite nanotubes (HNTs) [18], polydopamine coated HNTs [19], β-cyclodextrins modified HNTs [20] and amino-modified HNTs [21] were investigated as the effective carriers for HPW and created the additional proton transport pathways along the high aspect-ratio HNTs. The sulfonated poly(ether ether ketone) (SPEEK) membranes adding those fillers exhibited much higher proton conductivity than the SPEEK control membrane, and the HPW leakage was successfully slowed down or prevented in those membranes since the membrane conductivity remained almost constant for a long time under immersion in liquid water.

Furthermore, it has been demonstrated that, by introducing hybrid fillers with various dimensions, the formation of three-dimensional thermal/electrical conduction networks would improve the thermal/electrical conductivity of the composites [22,23,24,25]. For instance, carbon nanotubes (CNTs) and graphite sheets as hybrid fillers easily formed a continuous network, with the one-dimensional CNTs bridging the adjacent two-dimensional platelets to create additional pathways for heat flow [26]. Xie et al. prepared a thermal grease filled with 1 wt% graphene nanosheets and 63 vol% alumina particles, and the combination of hybrid fillers improved the thermal conductivity of the grease from 2.7 to 3.45 W m^−1^ K^−1^ [27].

Similar to heat or electron conduction, proton conduction might also benefit from the combined advantages of each filler. However, up to now, studies on the proton conductive membranes with hybrid filler consisting of nanotubes and nanosheets have not been reported. Therefore, in this work, sulfonated poly(ether ether ketone) (SPEEK) composite membranes filled with hybrid fillers, polydopamine (PDA) coated graphene oxide (DGO), and HNTs (DHNTs) that were anchored with HPW were prepared to construct three-dimensional proton transport networks, resulting in significant improvement in proton conductivity.

## 2. Experimental

### 2.1. Materials

SPEEK was obtained from the sulfonation of poly(ether ether ketone) (PEEK, P450F, Victrex, Lancashire, UK) according to our previous work [28]. HNTs (Golden Sunshine Ceramic Co. Ltd., Zhengzhou, China) were purified according to the literature [29]. GO was synthesized by the oxidation of graphite powder (Qingdao Golden Days Graphite Co., Qingdao, China) by a modified Hummers method [30]. Sodium hexametaphosphate, concentrated sulfuric acid, sodium hydroxide (NaOH), sodium nitrate (NaNO_3_), potassium permanganate (KMnO_4_), hydrogen peroxide (H_2_O_2_), tris(hydroxymethyl)-aminomethane hydrochloride (Tris-HCl, 99.0%), dopamine hydrochloride (DA-HCl, 99.0%), dimethylacetamide (DMAc), and HPW (99.0%) were bought from Innochem Technology Co., Ltd., Beijing, China. All materials were used as received unless mentioned otherwise.

### 2.2. Samples Preparation

**Scheme 1:**
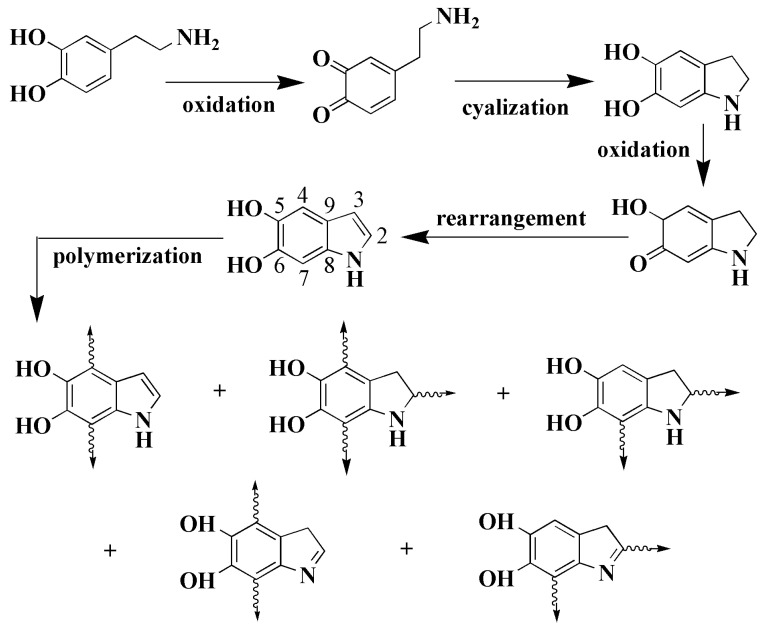
Polymerization mechanism behind the formation of PDA.

For the preparation of DHNTs and DGO, we used DA self-polymerization to form thin and surface-adherent PDA coating onto HNTs and GO [31]. Scheme 1 presents the polymerization mechanism behind the formation of PDA [32]. Specifically, 4 g HNTs or GO were ultrasonically (200 W, 40 kHz) dispersed in 100 mL of deionized (DI) water, and then tris-HCl (0.315 g) and DA-HCl (0.379 g) were added into the mixture sequentially, followed by an adjusting of pH to 8.5 (using NaOH). After stirring for 4 h, the mixture was centrifuged to retain the precipitate, which was repeatedly rinsed and centrifuged at least five times. The precipitate was first freeze-dried and then grounded to obtain DGO.

For the preparation of SPEEK/DGO/DHNTs (and SPEEK/DGO/DHNTs/HPW) composite membranes, SPEEK was dissolved in DMAc, and then DGO and DHNTs (and HPW) were ultrasonically (200 W, 40 kHz) dispersed in the SPEEK solution followed by magnetically stirring for 24 h. After degassing to remove air bubbles, the solution was solution casted (convection oven: 80 °C × 24 h; under vacuum: 100 °C × 12 h) to obtain the composite membranes (~60 µm thick). All the composite membranes were treated with sulfuric acid solution (1 mol/L) and washed by DI water sequentially before further characterization.

### 2.3. Characterization and Measurement

XPS characterization was performed by a PHI Quantera SXM X-ray photoelectron spectroscopy (XPS) (ULVAC-PHI, Miyazaki, Japan) with a 150 W monochromatic Al Kα radiation.

TGA analysis was carried out using thermogravimetric analyzer (TG-Q500, TA Instruments, New Castle, DE, USA) from 35 °C to 800 °C in the nitrogen environment at a heating rate of 20 °C /min.

XRD analysis was conducted by a diffractometer (D/Max-III C, Rigaku, Japan) with CuKa radiation operating at 40 kV and 200 mA [33,34,35] with a scan rate of 5.00°·min^−1^.

Morphological images were observed by scanning electron microscopy (SEM) (SU8010, Hitachi Co. Ltd., Tokyo, Japan) at an accelerating voltage of 5 kV. The samples were sputtered with gold before observation [36], and a certain selected area was scanned by an energy-dispersive X-ray spectroscopy (EDX) (X-Max20001, Horiba, Kyoto, Japan).

The ion exchange capacity (IEC) of a membrane sample was determined by the titration method [37] and calculated according to the following Equation:

(1) $$\mathrm{IEC}=0.01\times V_{\mathrm{NaOH}}{/m}_{\mathrm{dry}}$$

where m_dry_ is the dry weight of the sample, and V_NaOH_ is the volume of the NaOH standard solution (0.01 M) consumed during titration.

To determine the proton conductivity, membrane samples were placed in a custom built four-probe conductivity cell in liquid water (or in a humidity cabinet), and AC impedance measurement was performed on an electrochemical workstation (Zennium Pro., Zahner, Kronach, Germany). The proton conductivity (σ) of the membranes was determined according to the following Equation [38]:

(2) σ=L/(R×S)

where L is the distance between the two electrodes, R is the membrane resistance, and S is the cross-sectional area of the membrane.

The membrane samples were fully equilibrated in DI water or at various RHs before water uptake test. Water uptake of the membranes was calculated using the following Equation [39]:

(3) $$\mathrm{Water}\;\mathrm{uptake}\;=\;\frac{m_{w}{-\;m}_{d}}{m_{d}}\;\times \;100\%$$

where m_w_ and m_d_ are the wet and dry weight of the membrane, respectively.

## 3. Results and Discussions

### 3.1. Structure of DGO and DHNTs

**Figure 1:**
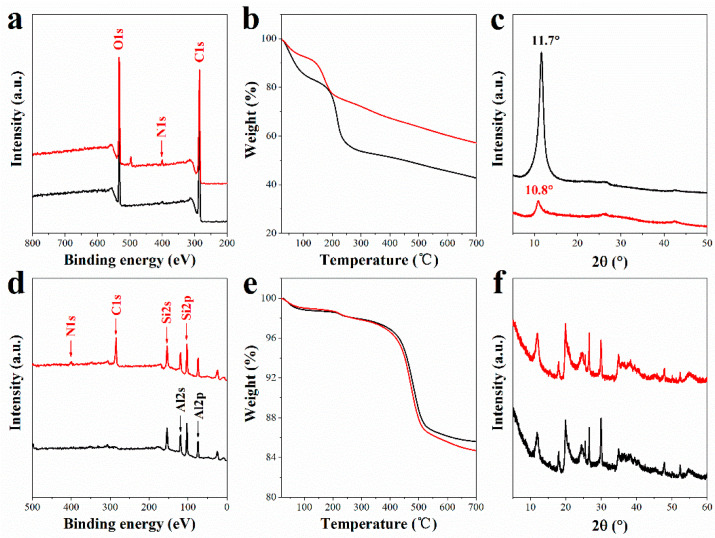
(**a**) XPS, (**b**) TGA, and (**c**) XRD curves of GO (black line) and DGO (red line). (**d**) XPS, (**e**) TGA, and (**f**) XRD curves of HNTs (black line) and DHNTs (red line).

XPS, TGA and XRD were used to verify the successful introduction of PDA on the surfaces of GO and HNTs, which could provide many hydroxyl groups and secondary amine groups to anchor HPW via hydrogen bond interactions and acid-base pairs, respectively. For the XPS survey curves as shown in Figure 1a,d, compared to GO and HNTs, both DGO and DHNTs show an additional characteristic N1s peak that originates from the PDA coating on filler surfaces. For the TGA curves in Figure 1b, the weight loss of GO at 200 °C was ~40 wt%, due to the evaporation of interlamellar water and the decomposition of surface oxygen-containing groups. In contrast, the weight loss of DGO was ~23 wt%. This phenomenon might result from the partial reduction of GO by PDA [40,41], which lowers the content of oxygen-containing groups on GO and improves the thermal stability of the nanosheets. The reduction of GO by PDA would also reduce the interlayer spacing, which is confirmed by XRD. As shown in Figure 1c, a sharp diffraction peak (2θ = 11.7°) was assigned to the 002 plane of GO with d-spacing of 0.95 nm. After adding PDA, the peak shifts to smaller angle (2θ = 10.8°), indicating the decrease of d-spacing [42]. As for HNTs, by the addition of PDA, no chemical reaction occurs between the nanotubes and PDA. Therefore, for HNTs and DHNTs, similar degradation behavior and the same XRD pattern are found as shown in Figure 1e,f, respectively. The slightly larger weight loss for DHNTs than that for HNTs is due to the additional degradation of PDA.

### 3.2. Performance of SPEEK/DGO/DHNTs Composite Membranes

**Figure 2:**
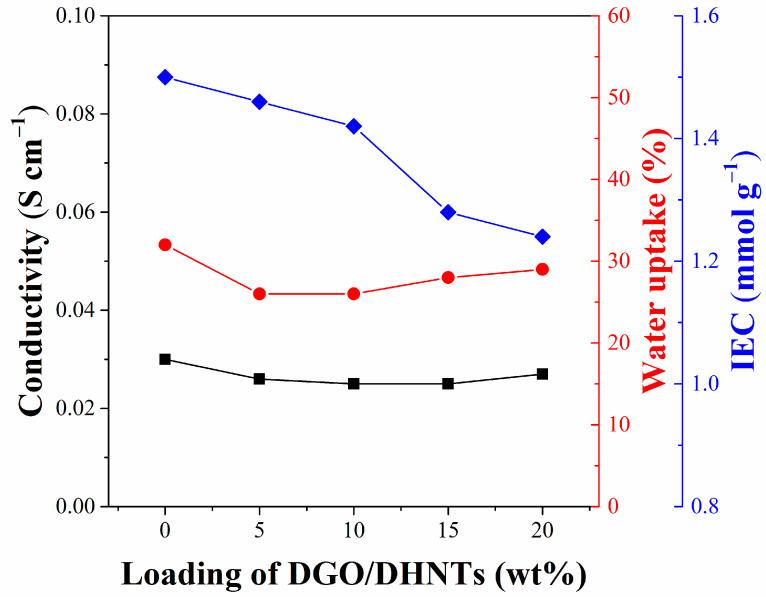
Proton conductivity, water uptake, and IEC of SPEEK/DGO/DHNTs composite membranes.

**Figure 3:**
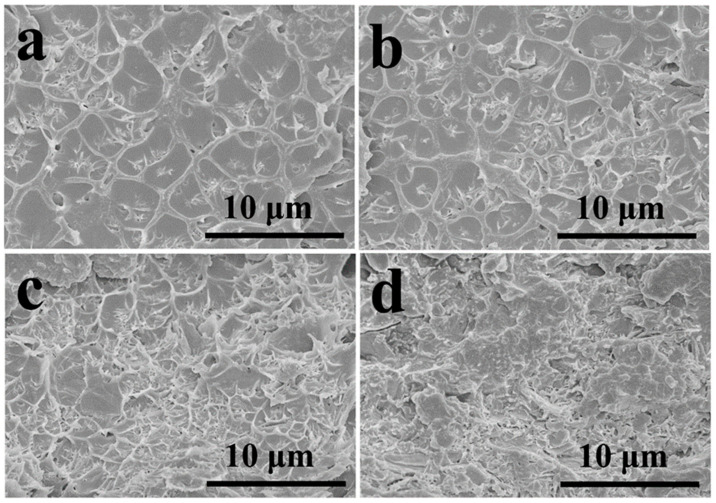
SEM images of SPEEK/DGO/DHNTs composite membranes with DGO/DHNTs (1:1) content of (**a**) 5 wt%, (**b**) 10 wt%, (**c**) 15 wt%, and (**d**) 20 wt%.

IEC, water uptake, and proton conductivity of SPEEK/DGO/DHNTs composite membranes are shown in Figure 2. The composite membranes exhibit a decreased IEC with the increase of DGO/DHNTs content, since DGO and DHNTs do not contain any ion-exchange groups showing very low IEC. Meanwhile, the formation of acid-base pairs between the basic secondary amine groups on PDA and the sulfonic acid groups in SPEEK also reduced the membrane IEC. However, the acid-base pairs, as well as the hydrogen bonding interactions, could also facilitate the dissociation of sulfonic acids to promote the proton transport in the composite membrane [18]. As a result, the proton conductivity of the composite membranes remains almost unchanged with the increase of DGO/DHNTs content. As for the water uptake, when a small amount of DGO/DHNTs is added, the acid-base pairs result in the decrease of water absorption of the membrane. However, due to the existence of many hydroxyl groups on the PDA coating, DGO and DHNTs would show very high intrinsic water absorption. Therefore, the water uptake of the composite membrane increased with a further increase of DGO/DHNTs content. Furthermore, the filler dispersion was observed by SEM. As shown in Figure 3, with the increase of DGO/DHNTs content, more aggregates are exposed on the fracture surface and more cracks are found, indicating the poorer dispersion of DGO and DHNTs.

### 3.3. Performance of SPEEK/DGO/DHNTs/HPW Composite Membranes

**Figure 4:**
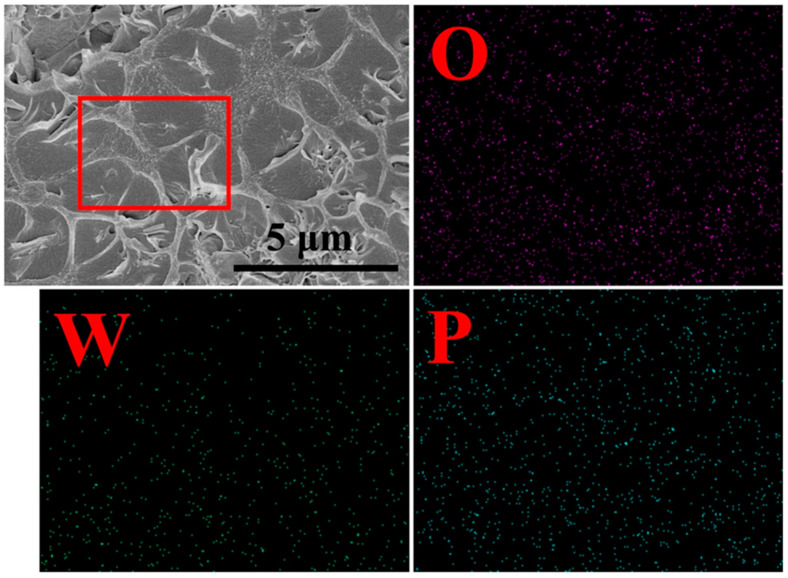
SEM-EDX mapping (element O, W and P) of SPEEK/DGO/DHNTs/HPW (90/5/5/60) composite membrane.

In this work, we aimed to anchor HPW by DGO/DHNTs, while the poor dispersion would harm the anchoring efficiency. Moreover, the optimal performance is achieved for the composite membrane at the 10 wt% DGO/DHNTs. Therefore, the SPEEK/DGO/DHNTs/HPW composite membranes with various HPW contents were prepared based on the SPEEK/DGO/DHNTs (90/5/5) composite membrane. It is found that the HPW (containing O, P and W) is uniformly dispersed in SPEEK matrix based on the SEM-EDX mapping of SPEEK/DGO/DHNTs/HPW (90/5/5/60) composite membrane (Figure 4).

**Figure 5:**
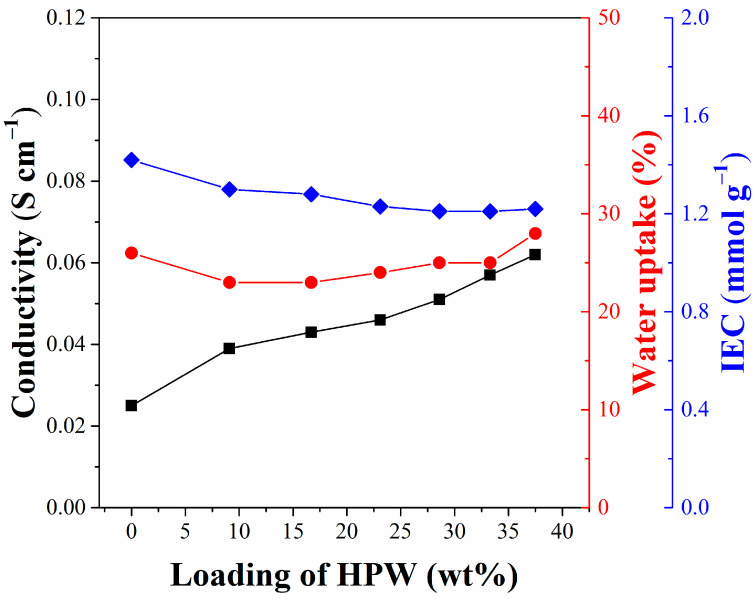
Proton conductivity, water uptake and IEC of SPEEK/DGO/DHNTs/HPW composite membranes.

**Scheme 2:**
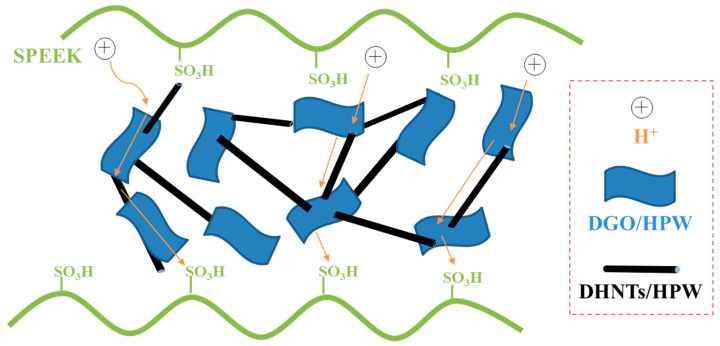
Proton conduction mechanism for hybrid three-dimensional proton transport networks.

As shown in Figure 5, the IEC of the composite membranes decreases with the increase of HPW content, which is due to the low intrinsic IEC of HPW (1.04 mmol/g) and the formation of acid-base pairs between DGO/DHNTs and HPW that could reduce the number of exchangeable protons during titration. The proton conductivity of the composite membranes shows an upward trend by adding HPW due to the following reasons: (i) HPW possesses better proton conducting ability than SPEEK; (ii) one-dimensional DHNTs and two-dimensional DGO are connected to anchor HPW, forming three-dimensional proton transport networks that provide sufficient transport pathways for protons (Scheme 2); (iii) both DGO and DHNTs can form acid-base pairs and hydrogen bond interactions with HPW and SPEEK to shorten the proton conduction distance. The highest conductivity was 0.062 S cm^−1^ for the SPEEK/DGO/DHNTs/HPW (90/5/5/60) composite membrane, ~248% higher than that of the composite membrane without HPW (0.025 S cm^−1^). For the membrane water uptake, the presence of HPW inhibited the water absorption of DHNTs and DGO through the formation of acid-base pairs. However, when further increasing the HPW content, the composite membranes exhibit increased water uptake owing to the strong intrinsic water absorption capacity of HPW. As a result, the water uptake of the composite membranes firstly decreased and then increased with the increase of HPW.

**Figure 6:**
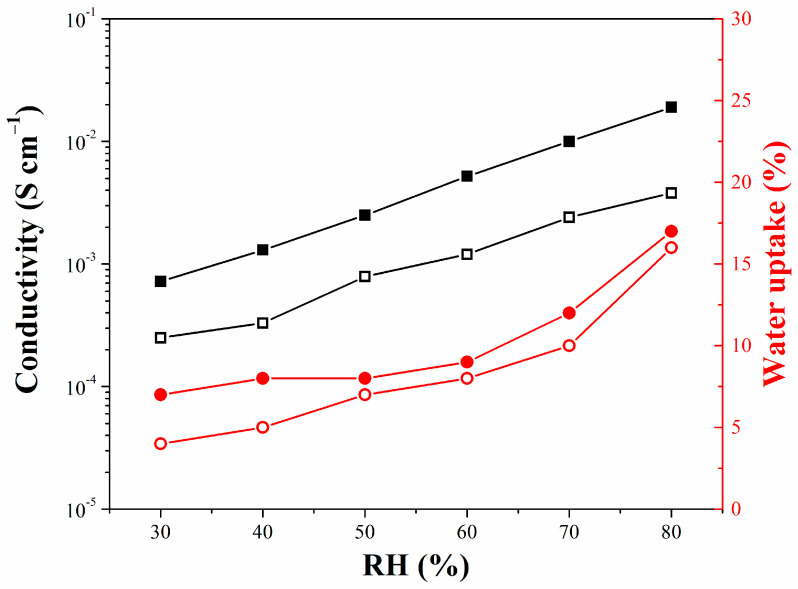
Proton conductivity and water uptake of SPEEK (hollow dots) and SPEEK/DGO/DHNTs/HPW (90/5/5/60) (solid dots) composite membrane at various RHs.

The proton conductivity and water uptake of SPEEK and SPEEK/DGO/DHNTs/HPW composite membrane at various RHs are shown in Figure 6. Owing to the excellent water-retaining capacity of HPW, the composite membrane exhibited higher water uptake than SPEEK membrane at RHs of 30~80%. The composite membrane also exhibited much higher conductivity than that of SPEEK membrane. On the one hand, the reserved water in the membrane could promote proton transfer. On the other hand, HPW is a strong acid, which also benefits the improvement of proton conductivity.

**Figure 7:**
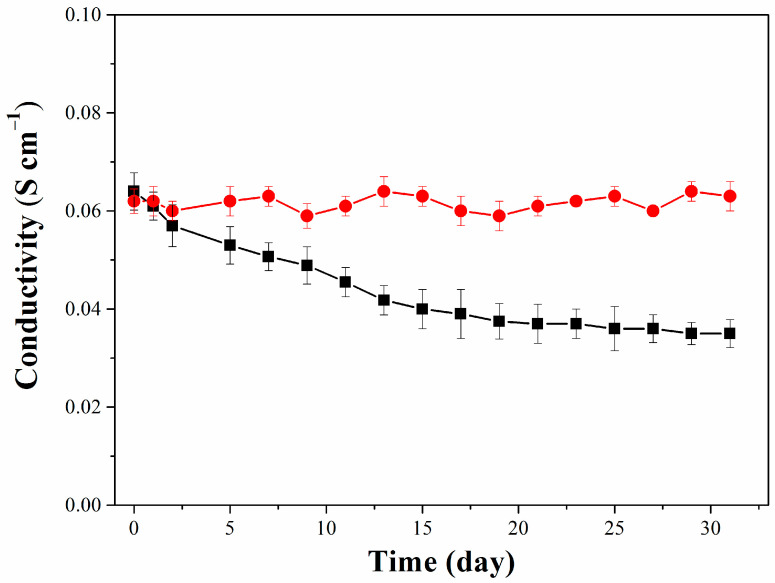
Proton conductivity of SPEEK/HPW (100/60) (black dots) and SPEEK/DGO/DHNTs/HPW (90/5/5/60) (red dots) composite membranes as a function of immersion time.

Moreover, the immobilization of HPW by DGO/DHNTs was confirmed via a membrane conductivity stability test, where the composite membranes with the same content of HPW were immersed in water that flowed continuously at 25 °C. As shown in Figure 7, the conductivity of the SPEEK/HPW composite membrane decreased rapidly, while that of the SPEEK/DGO/DHNTs/HPW composite membrane remains almost constant after 31 days, indicating the leakage of HPW has been successfully prevented.

## 4. Conclusions

In this work, we first introduced PDA on the surfaces of GO and HNTs, which was confirmed by XPS, TGA, and XRD, and then prepared the SPEEK/DGO/DHNTs/HPW composite membranes with various HPW contents based on SPEEK/DGO/DHNTs (90/5/5). It was found that HPW dispersed uniformly in SPEEK matrix. Although IEC decreased with the increase of HPW, the proton conductivity of the composite membranes showed an upward trend. The highest conductivity of 0.062 S cm^−1^ was achieved in the SPEEK/DGO/DHNTs/HPW (90/5/5/60) composite membrane, which exhibited higher water uptake and much better conductivity than the SPEEK membrane at low RHs. The conductivity of the SPEEK/DGO/DHNTs/HPW composite membrane remained almost constant during 31 days water immersion test, indicating the leakage of HPW has been successfully prevented. Therefore, our work provides a promising method to effectively anchor HPW in the composite PEMs with high and stable proton conductivity under low RH.

## Data Availability

Not applicable.
